# Self-Organizing Maps for Cellular *In Silico* Staining and Cell Substate Classification

**DOI:** 10.3389/fimmu.2021.765923

**Published:** 2021-10-29

**Authors:** Edwin Yuan, Magdalena Matusiak, Korsuk Sirinukunwattana, Sushama Varma, Łukasz Kidziński, Robert West

**Affiliations:** ^1^ Department of Applied Physics, Stanford University, Stanford, CA, United States; ^2^ Department of Pathology, Stanford University, Stanford, CA, United States; ^3^ Institute of Biomedical Engineering, Department of Engineering Science, University of Oxford, Oxford, United Kingdom; ^4^ Ground Truth Labs, Oxford, United Kingdom; ^5^ Big Data Institute/Li Ka Shing Centre for Health Information and Discovery, University of Oxford, Oxford, United Kingdom; ^6^ National Institute for Health Research (NIHR) Oxford Biomedical Research Centre, Oxford University National Health Service (NHS) Foundation Trust, Oxford, United Kingdom; ^7^ Department of Bioengineering, Stanford University, Stanford, CA, United States

**Keywords:** self-organization, *in silico* staining, e-pathology, cell subtype classification, segmentation

## Abstract

Cellular composition and structural organization of cells in the tissue determine effective antitumor response and can predict patient outcome and therapy response. Here we present Seg-SOM, a method for dimensionality reduction of cell morphology in H&E-stained tissue images. Seg-SOM resolves cellular tissue heterogeneity and reveals complex tissue architecture. We leverage a self-organizing map (SOM) artificial neural network to group cells based on morphological features like shape and size. Seg-SOM allows for cell segmentation, systematic classification, and *in silico* cell labeling. We apply the Seg-SOM to a dataset of breast cancer progression images and find that clustering of SOM classes reveals groups of cells corresponding to fibroblasts, epithelial cells, and lymphocytes. We show that labeling the Lymphocyte SOM class on the breast tissue images accurately estimates lymphocytic infiltration. We further demonstrate how to use Seq-SOM in combination with non-negative matrix factorization to statistically describe the interaction of cell subtypes and use the interaction information as highly interpretable features for a histological classifier. Our work provides a framework for use of SOM in human pathology to resolve cellular composition of complex human tissues. We provide a python implementation and an easy-to-use docker deployment, enabling researchers to effortlessly featurize digitalized H&E-stained tissue.

## Introduction

Cell organization in the tissue is deliberate (1–3) with specific cell types arranged into spatial structures driving tissue function both in health and disease, as well as patient outcome and therapy response in disease (2–5). Additionally, hospitals around the world routinely digitalize histological tissue slides collecting vast amounts of image data. Automatic extraction of clinical and actionable information from histological images is, therefore, the next aim in the field of digital pathology. However, automatic reading of digitized histological specimens is hindered by a complex nature of the images characterized by cellular and spatial heterogeneity, where both cell morphology and spatial distribution are parameterized by high dimensional space. While machine learning and, in particular, deep learning models applied to histopathology provide the first evidence that automatic slide reading might be possible (6–8), the narrow focus of these models on specific diseases or cell types (9) as well as their black-box nature hinders their interpretability and widespread use (10). Digital pathology thus needs more transparent models allowing for manual validation and broader application.

Parallel to computer vision methods, biochemical methods based on immunohistochemical tissue staining or RNA Sequencing (RNA Seq) can be used to resolve tissue architecture. Interestingly, spatially resolved multiplexed imaging was demonstrated to outperform conventional biomarker studies in predicting patient response to anti-PD-1/PD-L1 therapies (11). Biochemical techniques, such as immunohistochemistry, multiplex ion beam imaging by time of flight (MIBI-TOF) (12), or termed co-detection by indexing (CODEX) (13), are based on immune staining and require sophisticated reagents, lengthy staining optimizations, and specialized visualization equipment, and are thus unfit for large-scale datasets. Single-cell RNA Seq techniques like Drop-Seq (14) offer transcriptomic profiling at scale but entirely lose the spatial tissue context and do not accurately capture all cell types, as certain cell populations die because of tissue dissociation and other cells are too large to fit the microfluidics. These problems can be solved with new spatial transcriptome profiling platforms like Slide-seq (15), yet with a resolution tradeoff, as they can only profile small groups of cells. More importantly, both single-cell RNA seq and spatial transcriptomic platforms entirely lose information about cell morphology.

In this work, we leverage nuclear segmentation, self-organization algorithms, and non-negative matrix factorization (NMF) to automatically and quantitatively summarize nuclear morphology and spatial organization in the histological images. Our method, called Seg-SOM, performs nuclear segmentation, classification, and *in silico* nuclear labeling on the hematoxylin and eosin (H&E)-stained tissue section images routinely used in the clinic. Seg-SOM serves as a histological image dimensionality reduction method that provides descriptive H&E image statistics. The technique utilizes an artificial neural network called self-organizing map (SOM) (16) that learns to group nuclei by morphological features, such as nuclear shape and size, into a lower-dimensional space that is visually interpretable. We show that Seg-SOM can delineate major cellular lineages and reveal nuclear substates. We validate our approach using multicolor immunofluorescence and apply the workflow to two large breast cancer image datasets we generated to (1) predict lymphocyte infiltration and (2) devise spatial biomarker to classify breast ductal carcinoma *in situ* (DCIS) lesions into changes that presented alone or accompanied by invasive breast cancer. We anticipate our approach can facilitate a greater understanding of cell morphological and spatial dynamics in complex tissue environments, and their relationships with disease stage or other clinical correlates. Importantly, Seg-SOM opens the door to mining vast amounts of archival data and publicly available histological images without requiring additional staining or hand-labeled training sets. To that end, we have made our code publicly available. Modular implementation of our pipeline allows for easy-to-use application and extension to specific use cases.

## Results

### Seg-SOM Workflow: Segmentation, Self-Organization, Clustering, and Applications

Quantification of cell morphology is challenging because the visual features of each cell are parametrized by high dimensional space. The purpose of the Seg-SOM method is to transform the high dimensional visual features of each cell into an interpretable, lower-dimensional space where they can be used for *in silico* labeling of cells. In this manuscript, we focus on features pertaining to the morphological appearance of cellular nuclei.

**Figure 1:**
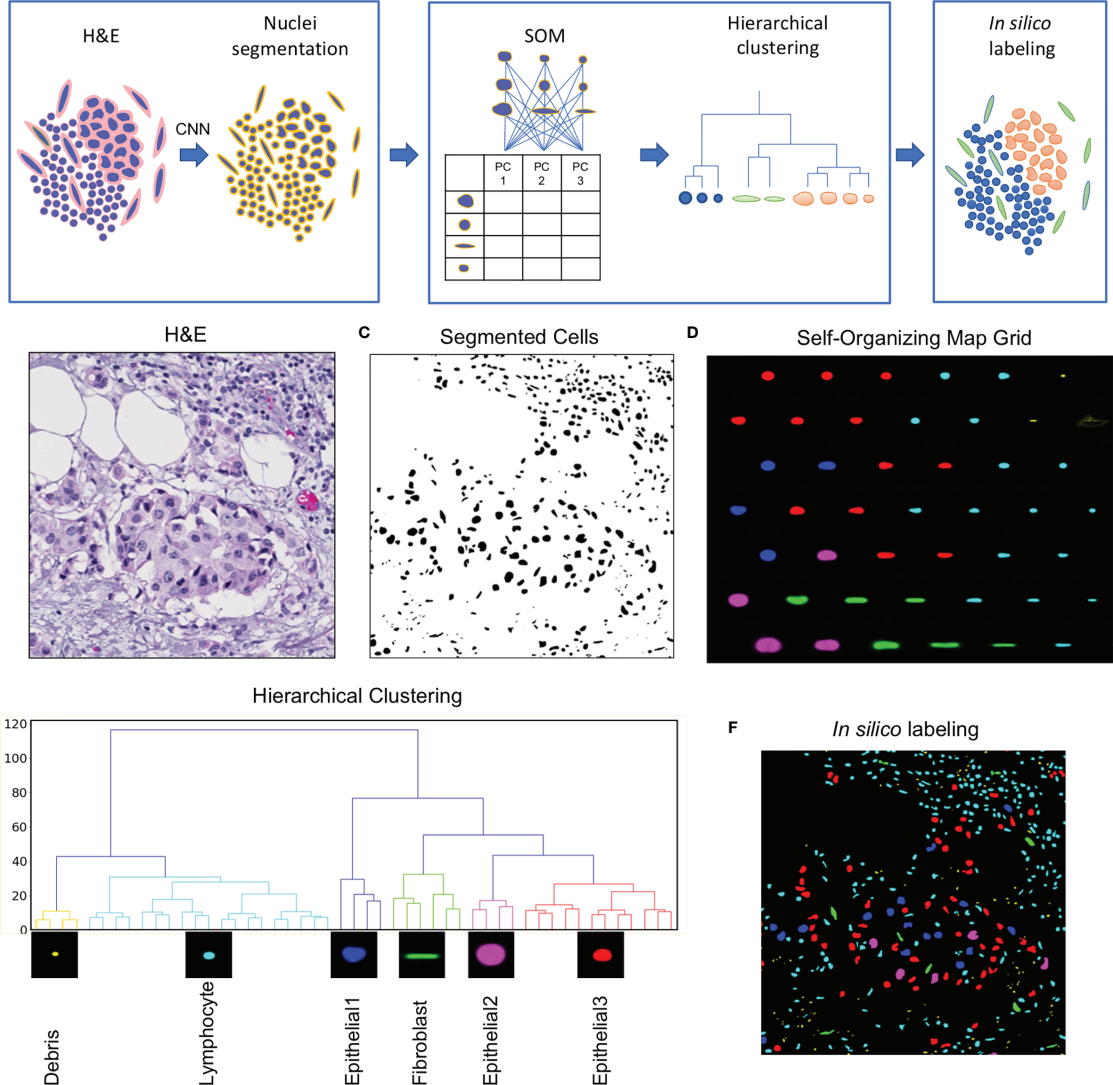
Seg-SOM pipeline reveals spatial nuclear organization in complex tissue images. **(A)** A schematic of Seg-SOM pipeline workflow. (1) Input H&E images are used for nuclear segmentation using a convolutional neural network (CNN). (2) SOM is trained on PCA feature vectors describing each nucleus in the dataset, and discovered SOM nodes are hierarchically clustered into classes. (3) Segmented H&E images are *in silico* labeled according to the hierarchical SOM class they belong to. **(B)** Standard hematoxylin and eosin (H&E) stained image showing a cluster of epithelial cells in the center, with a region of lymphocytic infiltration on the top right side. **(C)** Cellular nuclei segmented from an H&E input image in **(A)**, by the nuclei segmentation neural network; background indicated in white and nuclei indicated in black. **(D)** Segmented nuclei organized by the SOM into a 7×7 node grid. The learned axes arrange nuclear size in the vertical direction and nuclear aspect ratio in the horizontal direction. SOM nodes are colored by the classes learned by hierarchical clustering. **(E)** The linkage tree for the 49 SOM nodes, with six hierarchical clusters shown in different colors. The nodes with the smallest nuclei are themed Debris and colored yellow. The cyan-colored small and round nuclei nodes are called Lymphocyte. Nuclei of intermediate size, corresponding to colors red, blue, and pink, are called Epithelial1, Epithelial2, and Epithelial3. Long nuclei with a high aspect ratio, colored green, are annotated as Fibroblasts. **(F)** The segmented H&E image corresponding to **(B**, **C)**, *in silico* labeled by hierarchical SOM clusters, showing the concentration of Epithelial1, Epithelial2, and Epithelial3 nuclei in the center of the image (red, pink, and blue), and the Lymphocyte nuclei enriched in the top right corner.

The Seg-SOM pipeline consists of three parts: (1) nuclear segmentation (**Figure 1A, box 1**), (2) training of a self-organizing map (SOM) and hierarchical clustering of discovered nuclear subtypes (**Figure 1A, box 2**), and (3) *in silico* labeling (**Figure 1A, box 3**). The pipeline takes a standard H&E image as an input (**Figure 1B**). An input image is next fed into a segmentation neural network assigning every pixel in an input image as nuclei or nuclear border (**Figure 1C**). Subsequently, every nucleus is extracted from the segmented image and converted into a feature vector using PCA decomposition. The SOM is trained on a collection of the feature vectors, representing all nuclei present in the investigated dataset, learning to self-organize the nuclei into nodes on a user-defined grid (**Figure 1D**). During this self-organization process, each node on the SOM grid learns its own signature feature vector. As the SOM model trains successively on the PCA decomposed feature vectors of each segmented nucleus in the dataset, the elements of each SOM grid node’s feature vector are learned by the model automatically, through the learning and update algorithm. The adjacent nodes on a SOM grid are organized so that they constitute a continuum of nuclear shapes and visually resemble each other. We chose a 7×7 hexagonal SOM grid composed of 49 total cell nuclei nodes, which we found to be sufficient for representing the nuclear heterogeneity present in the training set. The learned SOM nodes (**Figure 1D**) display an organizational structure along the two major axes of the SOM grid, with the left-right axes organized by a large to small nuclear size gradient, and the top-bottom axes organized by nuclear aspect ratio.

We used hierarchical clustering of the SOM node feature vectors to group them into nuclear clusters (**Figure 1E**). We found that six clusters work well to differentiate between major tissue cell lineages that can be appreciated from the H&E staining: epithelial cells, fibroblasts, and lymphocytes. Based on the shape size and the spatial distribution in the tissue, we annotated each of the hierarchical clusters to further assist in the interpretation of the results. The small yellow matter grouped in SOM hierarchical class 1 (**Figure 1E**), with physical sizes between 0.1 and 2 µm, likely correspond to small bits of extracellular matter and are not real cell nuclei, and hence we term them “Debris.” We call the cyan-colored nuclei, grouped by clustering into class 2, Lymphocytes. The SOM hierarchical Lymphocyte class nuclei have sizes between 2 and 10 µm, are small, and nearly circular in shape (**Figure 1E**). The hierarchical SOM class 1, called here Debris, and class 2, called here Lymphocyte, form the first major branch of the clustering dendrogram (**Figure 1E**). The second dendrogram branch consists of (1) the larger, highly circular blue nuclei, measuring between 10 and 15 µm in diameter, which we annotated as “Epithelial1”; (2) the smaller, diversely shaped nuclei measuring between 10 and 12 µm in diameter of red nuclei are called “Epithelial3”; and (3) the large, relatively high-aspect ratio, pink nuclei, between 15 and 25 µm, which we called as “Epithelial2”. Hierarchical clustering of SOM nodes additionally identified a scattered population of cells with elongated nuclei that likely correspond to fibroblasts, which are labeled in green.

The learned grid of SOM nodes and the corresponding feature vectors can be used to digitally “stain” segmented nuclei on other images (**Figure 1F**). This is achieved by comparing the PCA feature vector of every nucleus on a new image to feature vectors to the trained SOM nodes and assigning the new nuclei to a SOM node with the smallest root-mean-square error. The nucleus in question is then colored by the hierarchical classification of its designated SOM node.

SOM grid can be extended to encompass other cell morphological features, such as cell color and texture. When including other visual features in the SOM grid, it may become desirable to extend the SOM model to higher dimensions. The spatial dimensionality of the SOM grid determines the number of axes of information that can be represented, and we have also trained four-dimensional SOM models that self-organize into axes of nuclear size, shape, texture, and color information (**Supplementary Figure 3**).

### Validation of SOM Clusters With Immunofluorescence Staining

**Figure 2:**
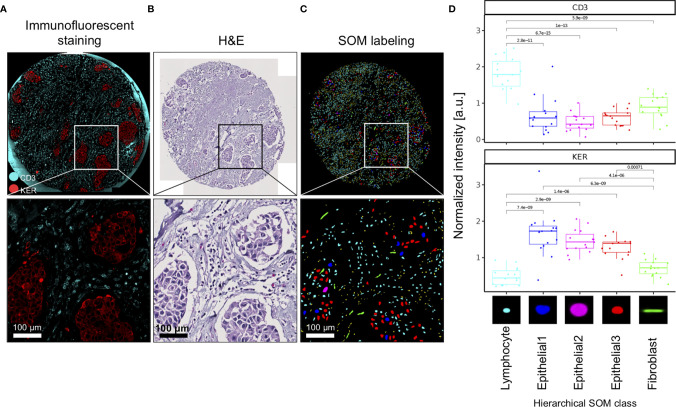
Validation of SOM *in silico* labeling with immunofluorescent staining. **(A)** A representative image of a TMA core and corresponding magnified regions: stained with CD3 (cyan) and KER (red), **(B)** H&E stained, and **(C)** *in silico* labeled with hierarchical SOM classes. Bottom panels of **(A–C)** are magnifications of the region boxed in the upper panel. **(D)** Mean CD3 and KER staining intensities of 15 IDC TMA tissue cores were shown for each of the SOM hierarchical classes. a.u., arbitrary units.

We used immunofluorescence staining to validate nuclei type annotations of clustered SOM nodes. CD3 was used to delineate T cells and pan-cytokeratin (KER) to delineate epithelial cells (**Figure 2A**). First, we show that the spatial pattern and density of the CD3-stained T cells (turquoise stain **Figure 2A**) corresponded to small, densely packed nuclei in the stromal compartment on the corresponding H&E image (**Figure 2B**), and the SOM hierarchical class was annotated as Lymphocytes (**Figure 2C**). Secondly, we show that spatial distribution and density of the epithelial cell clusters stained by KER (red stain **Figure 2A**) and arranged in apparent tumor island clusters on the H&E stain (**Figure 2B**) reflects nuclei labeled by hierarchical SOM classes annotated as Epithelial1, Epithelial2, and Epithelial3, and indicated by nuclei colored red, blue, and pink (**Figure 2C**). We further quantified the fluorescence staining intensity of each Seg-SOM hierarchical class (**Figure 2D**) by computationally collecting results from 15 breast cancer tissue microarray cores with breast invasive carcinoma *in situ* (IDC). Quantification of CD3 signal intensity around the nuclei shows that CD3 staining is significantly enriched in Lymphocyte SOM class compared to other hierarchical SOM clusters (**Figure 2D**, top). Furthermore, we show that SOM-labeled epithelial clusters have significantly higher KER staining intensity compared to Lymphocyte and Fibroblast SOM clusters (**Figure 2D**, bottom). Therefore, we have shown there is a strong correlation between the KER and CD3 normalized stain intensity and the Seg-SOM identified Epithelial and Lymphocyte class nuclei, respectively.

### Seg-SOM Estimates Lymphocyte Infiltration in the Tissue

**Figure 3:**
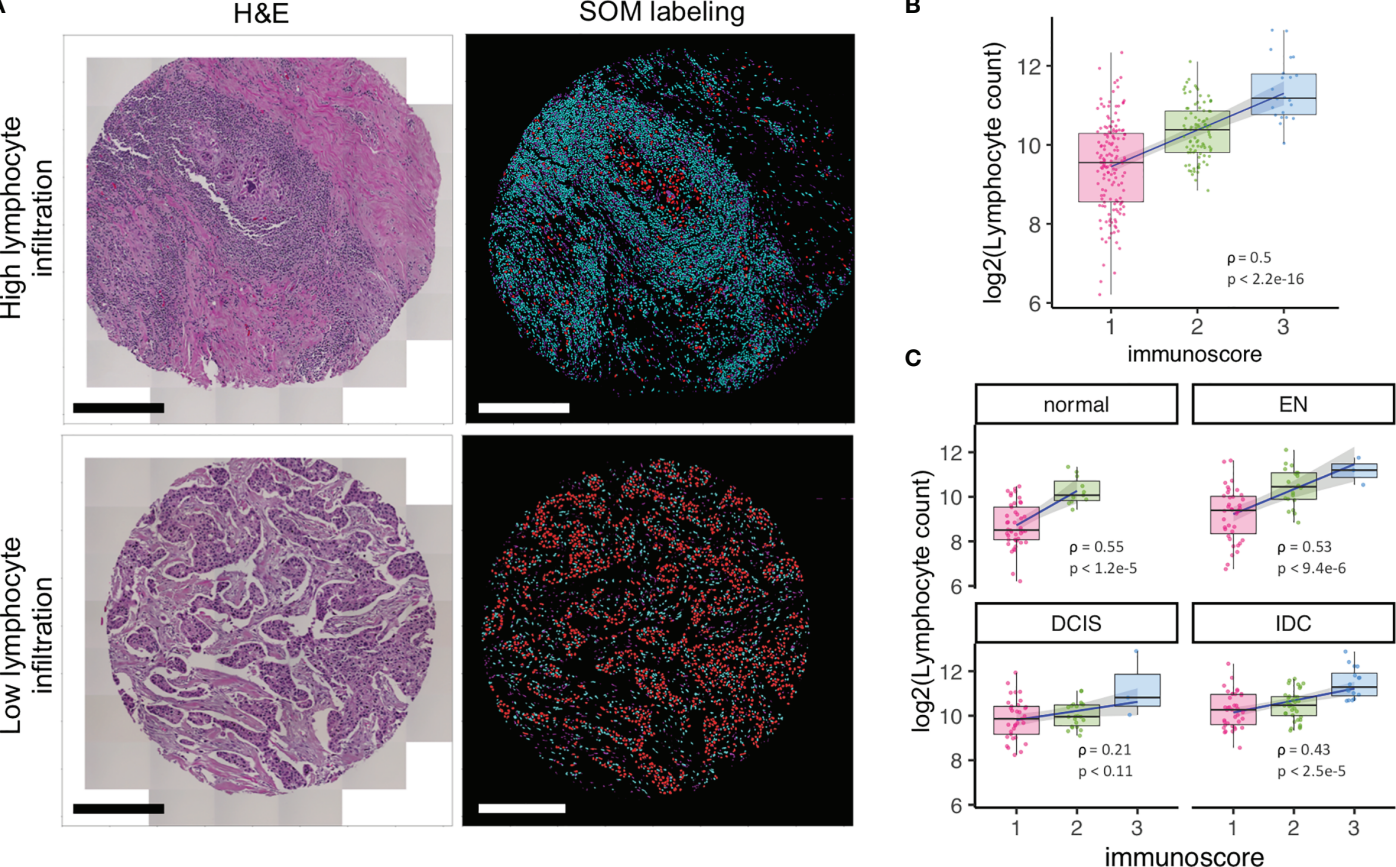
SOM *in silico* labeling can be used to predict lymphocyte infiltration in TME. **(A)** Left panel, H&E images of breast tissue TMA cores with high and low lymphocyte infiltration, respectively. Right panel, corresponding images of segmented nuclei *in silico* labeled with SOM hierarchical classes into either combined SOM Epithelial class (red) or SOM Lymphocyte class (cyan). **(B, C)** TIL infiltration assessed by a pathologist as an immunoscore (1-low, n = 155; 2-medium, n = 89; 3-high, n = 22), plotted against the number of SOM Lymphocyte class nuclei predicted by the Seg-SOM pipeline **(B)**, estimated on breast progression dataset composed of 285 breast TMA images combined and **(C)** stratified by different stages of breast cancer progression: normal breast ducts (normal, n = 56), ducts with early neoplasia (EN, n = 62), ducts with ductal carcinoma *in situ* (DCIS, n = 58), and regions of invasive ductal carcinoma (IDC, n = 90). Scale bars in **(A)** are 375 µm.

The tumor ecosystem is a complex mixture of transformed cancer cells and a variety of associated normal cell types like infiltrating immune cells, fibroblasts, endothelial cells, and other cell types (17). Tumor-infiltrating lymphocytes (TILs) reflect an adaptive immune response to tumors. TILs are well documented to determine therapy responsiveness (17) and are associated with favorable prognosis (18, 19). Nowadays, *in situ* lymphocyte infiltration evaluation is being increasingly suggested to be added as a new component to the traditional TNM (Tumor size, lymph Node spread, Metastasis presence) scoring (20–22). TIL estimation is also being used as an inclusion criterion to select patients for clinical trials or prescreen candidates for immunotherapy targeting T-cell responses like anti-CTLA4, anti-PD-1, and anti-PDL1 antibodies. We show that Seg-SOM labeling can serve to estimate lymphocytic infiltration. We applied the Seg-SOM method to a dataset composed of 266 tissue microarray core images of the subsequent stages of breast cancer progression including normal breast ducts (normal), breast ducts with hyperplasia/early neoplasia (EN), breast ducts involved with ductal carcinoma *in situ* (DCIS), and regions of invasive ductal carcinoma (IDC). We show how the Seg-SOM pipeline can identify hierarchical SOM Lymphocyte nuclei class (cyan) and combined Epithelial1, Epithelial2, and Epithelial3 hierarchical SOM class nuclei (red) (**Figure 3A**) on the representative H&E-stained tissue microarray cores with high and low lymphocyte infiltration. To quantify the accuracy of the Seg-SOM pipeline in estimating the lymphocytic infiltration, we asked whether the number of nuclei assigned as hierarchical Lymphocyte nuclei class corresponds to an infiltration score assessed by a surgical pathologist, which we call here an immunoscore. When considering all stages of progression, we find a moderately strong correlation (ρ = 0.5) between the number of Seg-SOM-annotated Lymphocyte nuclei and the immunoscore, at an extremely high confidence level (p < 2e-16) (**Figure 3B**). When examining the individual correlations between the number of hierarchical SOM Lymphocyte class nuclei and the immunoscore, stratified by breast tumor progression stage (**Figure 3C**), we find that the correlation is stronger for normal and EN tissue types (ρ > 0.5), compared to DCIS (ρ = 0.21) and IDC (ρ = 0.43). This could be due to greater morphological pleomorphism in the cancerous lesions, which leads to irregularly shaped epithelial nuclei being categorized as SOM hierarchical Lymphocyte class nuclei. Moreover, we show that the lymphocyte infiltration increases with tumor progression as the immunoscores and the number of Seg-SOM identified Lymphocyte class nuclei are higher in DCIS and IDC compared to normal breast ducts (**Supplementary Figure 1**). The calculated average immunoscores are 1.20, 1.42, 1.48, and 1.76 for normal, EN, DCIS, and IDC, respectively. The average hierarchical SOM Lymphocyte class nuclei counts per stage are 686, 1,150, 1,263, and 1,792, respectively. These results show that the Seg-SOM pipeline can be successfully used to estimate lymphocyte infiltration in complex images of histological tissue sections.

### Highly Interpretable Feature Extraction With Seg-SOM for Breast Cancer

**Figure 4:**
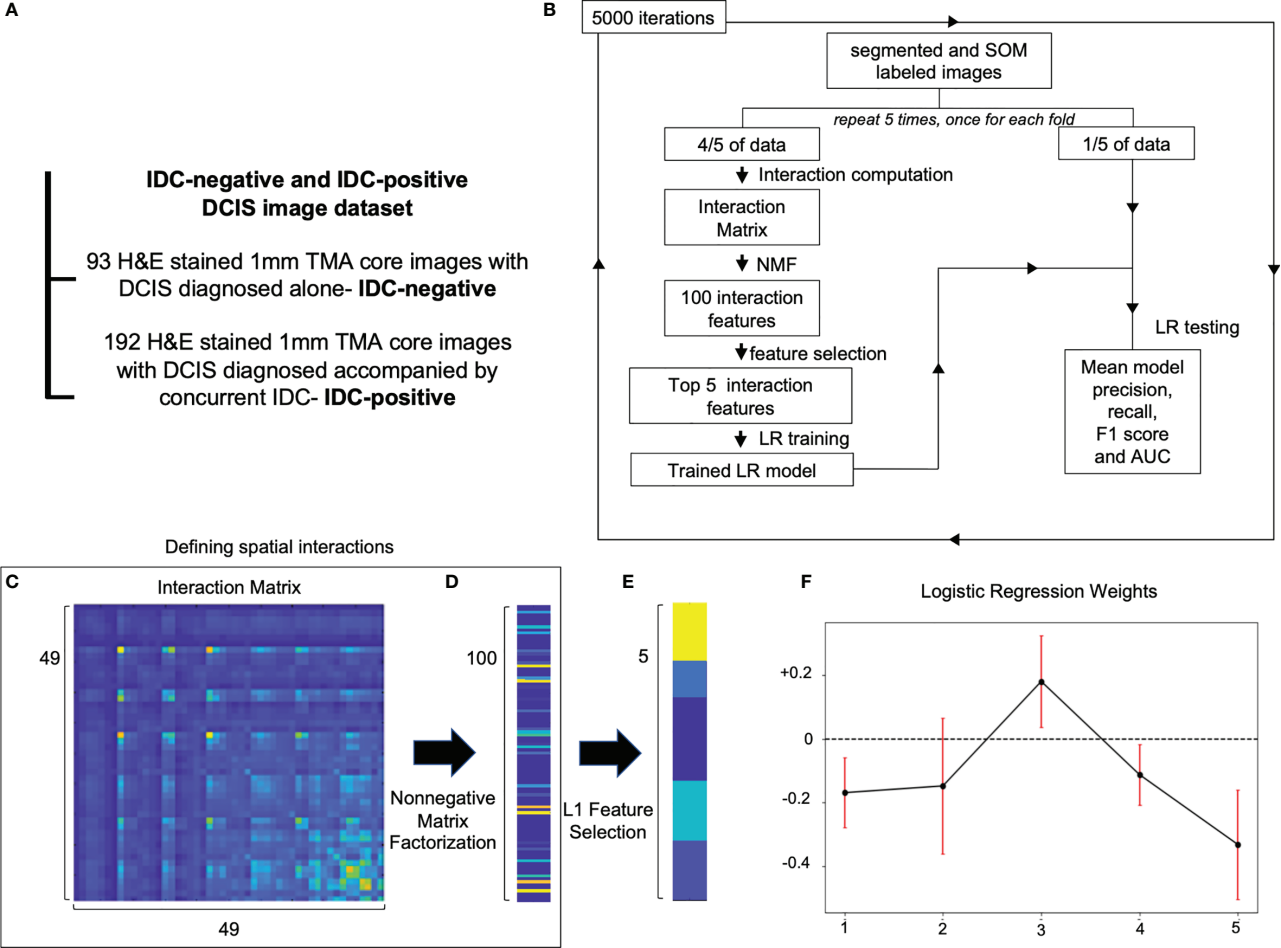
Spatial organization of SOM nodes predicts whether DCIS is likely to be IDC-negative or IDC-positive. **(A)** IDC-negative and IDC-positive DCIS dataset description. **(B)** A schematic representation of spatial feature extraction and IDC-positive/negative DCIS classification workflow. **(C–E)** Visualization of selection of top five spatial features; **(C)** an interaction matrix representing an average spatial distance between each of 49 SOM nodes. **(D)** 100 spatial features obtained by non-negative matrix factorization of the interaction matrix. **(E)** Top five spatial features with the largest magnitude of coefficients obtained with L1 Feature selection. **(F)** Top five logistic regression features weights obtained by 5,000 iterations of a five-fold cross-validation procedure used to classify IDC-negative and IDC-positive DCIS images. Red error bars indicate the standard deviation of the weights from 5,000 instances of five-fold cross-validation used to train the logistic regression model.

DCIS is a risk factor and a precursor lesion for IDC. Recent advances in contemporary cancer screening imaging techniques, like mammography, caused a significant increase in DCIS detection rates. However, studies show that only 13–52% DCIS patients do progress and develop IDC (23, 24). This raises concern that many DCIS patients get overtreated. Therefore, methods of improving DCIS stratification are urgently needed. We run the Seg-SOM pipeline on a dataset composed of 285 DCIS tissue microarray core images of samples from patients that were diagnosed with DCIS only (IDC-negative, n = 93) or patients diagnosed with DCIS with a concurrent IDC (IDC-positive, n = 192) (**Figure 4A**) to identify tissue architecture features predictive of whether DCIS is likely to present alone or accompanied by IDC. We hypothesize that IDC-negative DCIS tumor microenvironment (TME) differs from that of IDC-positive DCIS TME, and that tissue architecture features discriminating IDC-positive from IDC-negative DCIS lesions can reveal predictors of DCIS progression to IDC. The dataset is composed of pictures containing exclusively DCIS lesions, and thus we aimed to identify IDC-positive patients based only on the appearance of the DCIS.

Proximity of different cell subtypes in the tissue enables cell interactions and determines tissue fate. Each of the SOM nodes represents a distinct nuclear morphology and enables the discovery of nuclear subtypes and substates. Using the SOM grid trained on the IDC-negative/positive DCIS dataset, we constructed a set of features that characterize how different nuclear morphologies are spatially organized within the image. Specifically, we computed pairwise distances between cell nuclei representing each of the 49 SOM nodes and displayed the results using an interaction matrix (**Figures 4B, C**). The interaction matrix measures 49×49 elements, and each element of the matrix describes how likely nuclei from every two SOM nodes are spatially close to each other on the pictures of the dataset analyzed. Henceforth, we also use the term “interaction” to describe a scenario in which nuclei of one SOM node are spatially close to those of another node. We next reduced the dimensionality of the full interaction matrix to a 100-dimensional feature set using non-negative matrix factorization (**Figures 4B, D**). Subsequently, we trained an L1 classifier on the dimensionally reduced interaction matrix and extracted the top five features most predictive of DCIS being IDC-positive (**Figures 4B, E**). We next used the top five predictive interaction features to train a second L2 classifier (**Figures 4B, F**). We performed 5,000 iterations of the whole process, with five-fold cross-validation over the entire dataset (**Figure 4B**). Based only on the top five features, the classifier showed significant discriminative power to classify IDC-negative and IDC-positive DCIS pictures. It achieved an overall F1 score of 0.766 and AUC of 0.696 (**Table 1** and **Supplementary Figure 2**) in comparison to an F1 score of 0.744 and AUC of 0.779 achieved on the training dataset. The comparable F1 scores between training and test datasets indicate the model is not overfitting, and the higher AUC score on the training set indicates the prediction probabilities are better calibrated for the sample distribution of the training set.

**Table 1:** Results of logistic classification of IDC-positive *vs.* IDC-negative DCIS.

Precision	0.764
Recall	0.794
F1	0.776
AUC	0.696

The averaged precision, recall, F1, and area-under-the-curve (AUC) of 5,000 iterations of logistic regression model trained with five-fold cross-validation.

**Figure 5:**
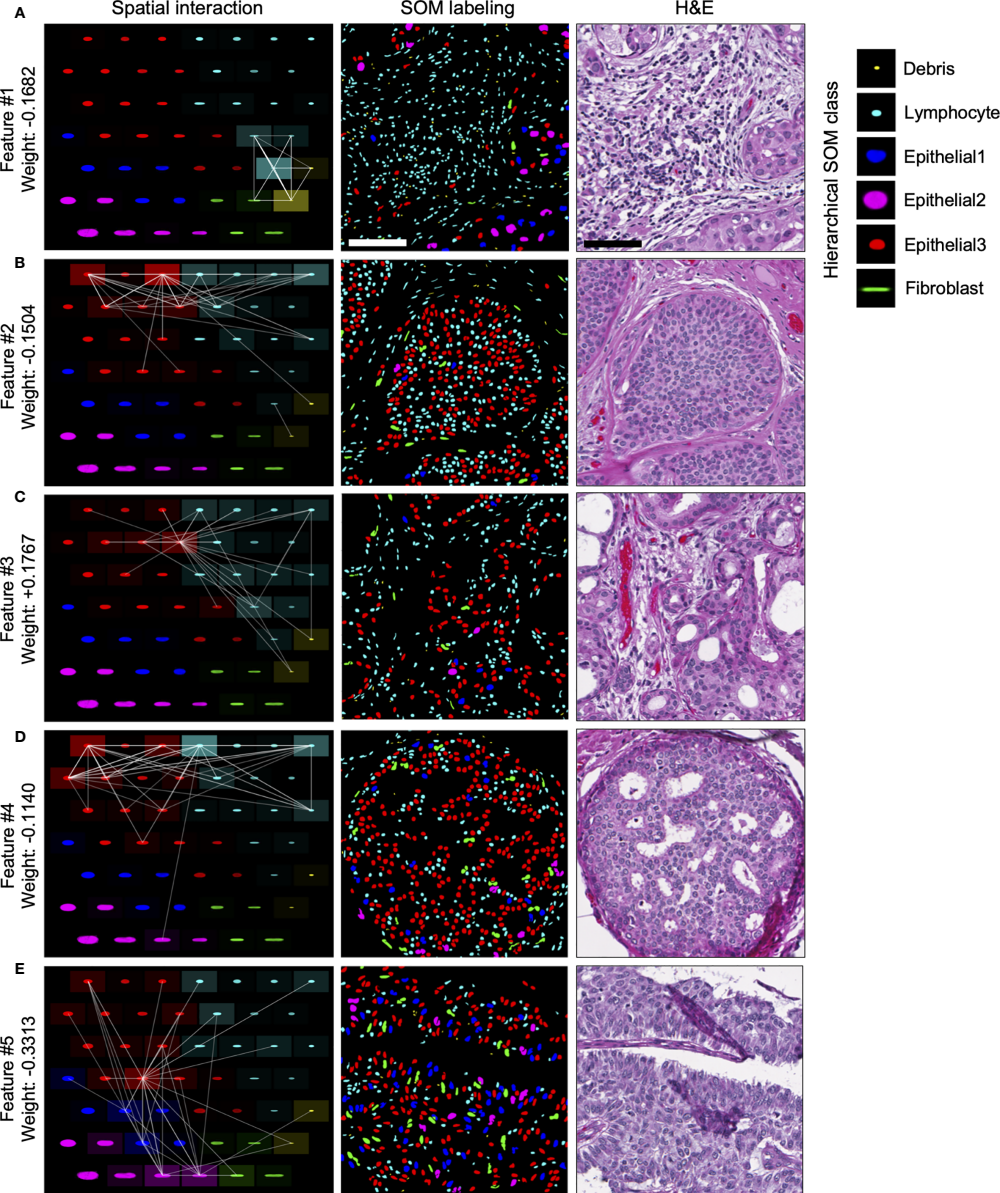
Visualization of top predictive features for IDC-positivity prediction in DCIS. **(A–E)** Left panel, visualization of the spatial interaction Features #1–5 in the context of the SOM node grid. Features #1–5 were learned during the IDC-negative IDC-positive classification task illustrated in **Figure 4**. The intensity of the white lines shows the interaction strengths between different SOM nodes in the given feature. The intensity of the tile surrounding each node shows the sum of all interactions with the node; in other words, it indicates how prominently the node contributes to the given feature. Middle and right panels, representative images of DCIS tissue regions enriched in Features #1–5. Middle panel, segmented and *in silico* SOM-labeled cell nuclei, colored by the hierarchical SOM class. Right panel, corresponding H&E images. Scale bars have 200 µm.

Of the top five selected model features, four features are negatively weighted, predicting the patient whom DCIS was imaged is more likely to be IDC-negative, while a single feature, Feature #3, is positively weighted, predicting a greater likelihood of the patient being IDC-positive (**Figure 4F**). We visualized the top five predictive spatial interaction features in the context of the SOM grid (**Figures 5A–E** left panel) and present them next to an example of DCIS region enriched in each feature, colored by SOM hierarchical classes (**Figures 5A–E** middle panel), and H&E staining (**Figures 5A–E** right panel). The five top features are visualized on the SOM grid using rectangles and lines highlighting and connecting interacting SOM nodes. The color of the rectangle indicates the hierarchical SOM class of the node. The brightness of the rectangle surrounding each SOM node indicates its total weighted contribution to the interaction feature and reflects the abundance of that node’s nuclear type. The brightness of the white node-connecting lines indicates the strength of interactions between SOM nodes. Feature #1 indicates lymphocyte infiltration, as it highlights interactions between a few hierarchical Lymphocyte class nuclei with hierarchical SOM Fibroblast class nodes (**Figure 5A**). Features #2 and #4 are enriched in larger, epithelial nuclei, primarily from the first row of the SOM grid (**Figures 5B, D**). Features #2 and #4 display overlap of SOM nodes and have a relatively large correlation coefficient of 0.2. Feature #3 characterizes a different epithelial nuclear SOM node, which the model associated with an increased likelihood of IDC-positivity. In spite of rather subtle differences in the nuclear shape of nuclei enriched in Feature #3, and those enriched in Features #2 and #4, the model indicates significant differences in the predictive power of these nuclei’s distribution in IDC-negative *vs* IDC-positive DCIS pictures. Compared to Features #2 and #4, Feature #3 describes interactions of cells all based around a single SOM node with an increased lymphocyte involvement (**Figure 5C**). Additionally, Seg-SOM labeling shows that Feature #3 (**Figure 5C** middle panel) displays a different spatial organization with lower cell density compared to Features #2 and #4 (**Figures 5B, D** middle panel). Finally, Feature #5 is enriched in a set of epithelial cells with elongated and larger nuclei (typically of 12 × 24 µm) colored purple and blue by the hierarchical clustering scheme. (**Figure 5E**). The model weight of Feature #5 is strong, at −0.33, and its negative leaning influences the classification well beyond its error bar, indicating its robustness. The negative model weight of Feature #5 is consistent with previous findings and clinical scoring practice (25) where nuclei with a long aspect ratio are typically associated with low-grade tumor cells and a lower chance of IDC progression.

In summary, our analysis of spatial nuclear distribution between different SOM-discovered nuclear subtypes disclosed a complex system of cell interactions predictive of whether DCIS is likely to be diagnosed alone or accompanied by the IDC in breast cancer patients. We revealed four nuclear subtype interaction patterns associated with DCIS lesions that are indicative of lower risk of progression from DCIS to IDC, and one epithelial/lymphocyte interaction pattern that correlates with an increased risk of DCIS to be accompanied by IDC.

## Discussion

We describe a computer vision method, Seg-SOM, that can discover nuclear subtypes in complex histological images based on their morphology. We use it to visualize nuclear heterogeneity and tissue architecture and thus reveal information that traditionally requires immune-staining and/or professional histological assessment. The Seg-SOM method operates on H&E stains routinely used in clinical practice. The Seg-SOM workflow consists of three primary steps: (1) nuclear segmentation, (2) nuclei grouping based on their shape and size performed by the self-organizing map algorithm combined with hierarchical clustering of SOM nodes, and (3) *in silico* cell type staining. SOMs have been previously used in digital pathology for red blood cell classification (26), megakaryocyte subtypes clustering (27), and analyzing 3D cell surface information (28). This work is the first to present the combination of SOMs and NMF as a general tool for dimensionality reduction of nuclear morphology, the grouping of nuclei in complex tissues, discovering nuclear subtypes, nuclear *in silico* labeling, and extracting machine learning features as potential spatial biomarkers.

In this work, we perform nuclear segmentation on H&E-stained tissue images, yet tissue images of any nuclear stain can be used as input to the Seg-SOM method. We further note that while we used a convolutional neural network (CNN) for nuclear segmentation, any other cell segmentation technique can be used.

We validated our annotation of hierarchical SOM nuclei classes and the *in silico* cellular staining by comparing them with immunofluorescent staining for markers of epithelial cells and lymphocytes, KER, and CD3, respectively. We did not expect Seg-SOM labeling to identically reproduce the results of immunofluorescent staining, as the Seg-SOM organizes cells only based on the shape of their nuclei, while immunofluorescence, used for the annotation validation, indicates the cytoplasmic protein expression. However, we show significant enrichment of CD3 IF staining around hierarchical SOM Lymphocyte class compared to hierarchical SOM Epithelial and Fibroblast classes, and enrichment of KER IF staining around hierarchical SOM Epithelial1, Epithelial2, and Epithelial3 classes compared to hierarchical SOM Lymphocyte and Fibroblast classes. This result demonstrates that Seg-SOM can be reliably used to *in silico* stain H&E images.

Furthermore, applying the Seg-SOM pipeline to two breast tissue image datasets, we demonstrate two applications of the Seg-SOM method. First, using *in silico* labeling, Seg-SOM allowed us to highlight tumor-infiltrating lymphocytes (TIL) and estimate TIL infiltration in normal and cancerous breast tissue. Secondly, we demonstrate how SOM nodes can be used to construct interpretable features of nuclear spatial organization for machine learning tasks. We show how analysis of the spatial distribution and proximity of different SOM-identified nuclear subtypes can be used for the classification of IDC-negative and IDC-positive DCIS images. Our approach can be especially valuable for identifying interactions between two cell populations and their prognostic value. We revealed that interaction of subtly different nuclear shape subtypes with distinct stromal cell subsets can contribute very differently to the likelihood of IDC concurrence. Specifically, we found two features describing nuclear interaction in DCIS, Features #3 and #4, that are enriched in epithelial nuclei of very similar shape and size, yet with an opposite likelihood of IDC-positivity. Our model also predicted that Feature #5, enriched in elongated nuclei, has a relatively strong predictive power of IDC-negativity. This finding is consistent with the fact that enrichment of long-aspect ratio cells characterizes low-grade tumors with a lower chance of progression.

There are important limitations to this study. First, Seg-SOM relies on the quality of images and segmentation performance. If the H&E staining of an analyzed image was significantly different from the images that the Seg-SOM segmentation model was trained on, one would expect an increase in segmentation errors. Certain debris on darker stains can be segmented as nuclei, and improperly stained nuclei may not be picked up. The quality of the nuclei segmentation can have a significant impact on subsequent steps of cell clustering and labeling by Seg-SOM, especially if the segmentation error is heterogenous amongst different cell types. Second, Seg-SOM may incorrectly assign classes to nuclei with changed morphology (due to cell division, cell death, or technical artifacts). In tumors with high nuclear pleomorphism like DCIS and IDC, dying tumor nuclei shrink and start to resemble smaller cells’ nuclei like lymphocytic nuclei. In our analysis, we report dying tumor cells that are stained with KER but were assigned by the Seg-SOM pipeline as hierarchical SOM Lymphocyte class (**Figure 2C**). The frequency of similar errors will vary and depend on tissue/disease characteristics. If the shape and size of the nuclei with changed morphology are similar to other nuclear classes, immunofluorescent staining is the only way to verify the nuclear identity. On the other hand, highly irregular nuclei get assigned to separate SOM grid bins and can be sorted out by the user. Moreover, SOM identified hierarchical Lymphocyte class likely includes NK, NKT, and B cells apart from CD3-positive T cells.

Despite these limitations, Seg-SOM can perform dimensionality reduction and *in silico* labeling of complex tissue images, containing thousands of different cells, within seconds, while relying on no tunable parameters. Our pipeline further has the advantage that it is entirely automated, does not require human labeling substantial training data or hand-crafted features sets, and is thus easily scalable, allowing for full standardization of the technique. The Seg-SOM workflow can be used unchanged to characterize different nuclear subtypes distribution in complex tissue environments using images of H&E-stained tissue. Importantly, the SOM model we present in this work can be retrained to discover new cellular subtypes in different tissue types and pathologies. The pipeline can be further modified to organize the morphology of whole cells if whole cell segmentation is used.

We anticipate that Seg-SOM will have a variety of uses in the digital pathology era. In particular, as a dimensionality reduction, segmentation, and *in silico* cell-type labeling tool, Seg-SOM provides the first and necessary step in exploring cellular heterogeneity and special cell organization in complex tissue environments, and importantly cell relationships with disease stage or other clinical correlates. Our approach lends itself to high levels of interpretability and can facilitate the discovery of prognostic and predictive markers associated with cell morphology and cellular interactions. We foresee Seg-SOM as a valuable discovery tool that opens doors to mine vast amounts of archival data and publicly available histological images.

## Methods

### Used Datasets Description

1. U-Net Training dataset: a publicly available dataset of 21,623 hand-annotated nuclear boundaries from 30 whole slide images that were H&E stained and captured at 40× magnification (29). The images were downloaded from The Cancer Genome Atlas (TCGA) and contained tissue from seven organs: breast, liver, kidney, prostate, bladder, colon, and stomach; including normal and tumor regions. Each whole slide image was cropped to 1,000 × 1000 images containing regions dense in nuclei and annotated using Aperio ImageScope software.
2. Immunofluorescence validation was performed on a set of 15 IDC tissue microarray cores of 1 mm in diameter. TMA was first H&E stained and scanned at 40× magnification. Next, the same TMA slide was decoversliped and immunofluorescently (IF) stained with pan-Cytokeratin, CD3, and DAPI. Images of IF-stained TMA were acquired by scanning each stain separately at 40× magnification. Therefore, each TMA core has four corresponding stain images: H&E, pan-Cytokeratin, CD3, and DAPI.
3. The breast cancer progression dataset used to estimate lymphocytic infiltration is composed of a 266 images of TMA cores stained with H&E including 56 normal breast tissue cores, 62 early neoplasia (EN) tissue cores, 58 cores with ductal carcinoma *in situ* (DCIS), and 90 cores with invasive ductal carcinoma (IDC). Each TMA core is 1 mm in diameter and was scanned at 40× magnification.
4 .IDC-negative and IDC-concurrent DCIS dataset used for extraction of SOM-derived classifier features is composed of 285 images of H&E-stained TMA cores containing 93 images of DCIS from a patient that was IDC negative and 192 images of DCIS from patients diagnosed with concurrent DCIS and IDC at presentation. Each TMA core is 1 mm in diameter and was scanned at 40× magnification.

### H&E Staining

TMA FFPE sections slides were first baked at 60°C for 16 h and subsequently deparaffinized and rehydrated using sequential incubation in Xylene, decreasing concentration of EtOH (100, 95, and 70%) and H2O. Next slides were incubated in hematoxylin for 1 min, washed with H2O, dipped 6× in bluing solution, washed with H2O, incubated in eosin for 2 min, and dehydrated using an increasing concentration of EtOH and Xylene. Slides were coverslipped and imaged at 40× magnification using a Leica Ariol slide scanner.

### Segmentation of Complex Tissue Images

The U-Net deep-neural network was used for nuclei segmentation from the H&E images. U-net separates the image into overlapping 256×256-pixel tiles. Each pixel within each tile is given a probability of belonging to one of three classes, either background, cell nuclei, or boundary between nuclei and background. In the final segmented image, each pixel is given the label of the class with the maximum probability. The U-Net was trained on a publicly available dataset of 21,623 hand-annotated nuclear boundaries from H&E images acquired at 40× magnification and taken from seven different organs (29). The U-Net network architecture is presented in **Supplementary Table 1**. Cell nuclei boundaries are weighted according to the scheme described in the original U-Net publication (30), teaching the model to focus more of its attention on discriminating cell boundaries. Without this weighting, we find that the U-net does not perform as well in segmenting regions of densely packed cell nuclei. The probabilities of all tiles in the image are merged, with overlapping portions merged according to the following formula: (1-d) (p2) + (d) (p1). The final cell nuclei boundaries can be used to extract the set of all pixel objects, representing individual nuclei, enclosed by a closed-looped boundary.

### Self-Organization of Segmented Nuclei

For this manuscript we trained SOM separately on two datasets: (1) breast progression dataset and (2) IDC-negative and IDC-concurrent DCIS dataset. For each dataset, after segmentation, each individual nuclei were cropped to an image size of 170×170 pixels (0.20 microns per pixel), with cell nuclei larger than 170×170 pixels excluded from further analysis. All cell nuclei were then rotated so that their major axes align. This was done by considering each pixel in the 170×170 crop of each cell as a separate two-dimensional data point (with coordinates on the x and y axis) and performing PCA on all pixels in the cropped image. The entire cropped image was then rotated by the angle of the resulting 1^st^ principal component axis, and the image is re-cropped to 170×170 pixels rotated crop. The resulting dataset consisted of N-cropped nuclei images of size 170×170, which can also be considered vectors of length 28,900 (170*170 = 28,900). PCA was subsequently used again to reduce the dimensionality of the dataset from vectors of 28,900 to a lower dimension D. We chose D = 1,000, which captured more than 98% of the variance in the data and allowed for accurate reconstruction of the original cell images. Additionally, we noted that the second PCA step allows faster SOM training. The training set of N vectors of length D was shuffled before SOM model training.

Before SOM model training, we initialized the SOM as a 7×7 hexagonal grid of random normal vectors *w* of dimension D = 1,000, where each vector is called a node, with H = 49 nodes in the resultant SOM grid. Using a larger H value sorts the dataset into finer-grained bins or SOM nodes. Traditionally, SOMs are fit to either hexagonal (six nearest neighbors) or rectangular grids (four nearest neighbors). Previous studies have indicated that hexagonal structures may be superior for visualization and fitting of data (31), due to the higher nearest neighbor connectivity.

We trained the SOM model for 100 epochs using MATLAB 2017b’s self-organizing map toolbox. During each timestep, *t* of each epoch vector *v* from the training set are iteratively chosen without replacement and compared to the vectors *w* of initiated SOM grid nodes. Each training vector *v* is being assigned to the SOM node that minimizes the Euclidean distance metric L(*w,v*) between SOM vector *w* and the training vector *v*. All nodes *w* of the SOM next are updated to become more similar to the training vectors *v* assigned to them, but to an extent that depends on a function G(*w,v*) that depends on the distance on the SOM grid between vectors *w* and the training *v.* As we are using a hexagonal grid, each node has six closest neighbors. The update formula is the following:

*w*(t) = *w*(t − 1) + G(L(w,v)) * a(t) * (*v* − *w*(t))

where a(*t*) is a monotonically decreasing function that can depend on both the timestep and the epoch of training. This way the SOM nodes “self-organize” into learned node vectors which are representative of the spectrum of cell nuclei shapes and sizes within the training set. The two dimensions of the SOM grid reflect nuclear size, along the horizontal axis, and nuclear aspect ratio, along the vertical axis.

### SOM Nodes Clustering

For each dataset, we performed hierarchical clustering of the trained SOM node vectors. The hierarchical clustering was performed in Python, using functions linkage and fcluster from the scipy.cluster.hierarchy module. The linkage function was called on the SOM vectors from each node, with method=‘ward’. The linkage was subsequently used as input to the fcluster function with criterion=‘maxcluster’ and six designated clusters. We tested various numbers of clusters and empirically found that six clusters identified interpretable groups of major tissue cell lineages, such as epithelial, lymphocyte, and fibroblast cells, for each cluster of SOM nodes.

### Immunofluorescent Staining

Slides that were stained with IF after H&E staining were first decoverslipped by incubation in Xylene. Next, antigen retrieval was performed by cooking slides at 95°C for 5 min in EDTA solution pH 9 in a pressure cooker. Subsequently, slides were washed with PBS, blocked with horse serum, and incubated 1 h with pan-Cytokeratin ab (1:1800, AE1/AE3, Novus) and CD3 ab (1:400, A045229-2, Agilent). Slides were washed and incubated with secondary goat antimouse AlexaFluor647 (1:100, A32728, Thermo Fisher Scientific) and goat antirabbit AlexaFluor555 (1:100, A32732, Thermo Fisher Scientific) for 30 min. Slides were washed and mounted in ProLong™ Gold Antifade Mountant with DAPI (P36931, Thermo Fisher Scientific). Stained slides were imaged using 40× magnification using Leica Ariol slide scanner.

### Comparison of Seg-SOM With Immunofluorescent Staining

Quantification of the intensity of immunofluorescence staining with pan-cytokeratin and CD3 was computed for hierarchical Seg-SOM classes to validate their predicted membership to major cell lineages. First, we spatially register the DAPI images of each core with their corresponding H&E image, by spatial cross-correlation of the intensities of the H&E and DAPI images. We then mapped the segmented nuclei to the IF image to quantify stain intensities. Cytokeratins and CD3 are localized in the cell cytoplasm; therefore, we considered the region around each nucleus to quantify the staining intensity. We dilated each nucleus with a kernel of 3×3 pixels for eight iterations for larger epithelial cells and five iterations for smaller lymphocyte cells to create a ring-shaped region that we would consider belongs to a cytoplasmic region of each cell. We computed stain intensity as the mean of the fluorescence signal within the ring-shaped region for every cell. In **Figure 3C**, the normalized staining intensity is defined as the final stain intensity for each cell divided by the average stain intensity for all cells in the image.

### Lymphocyte Infiltration Estimation

Based on the nuclear size and shape, hierarchical SOM clusters were further assigned to four clusters representing epithelial cells, fibroblasts, lymphocytes, and debris. Next, the Lymphocyte class cells were counted on every image of a breast progression dataset. In parallel, lymphocyte infiltration was scored by a surgical pathologist on a scale from 1 (low or no lymphocytic infiltration) to 3 (high lymphocytic infiltration) (**Figure 3A**, left side). Finally, the ggplot2 R package (32) was used to plot the log2 transformed count of Seg-SOM-labeled lymphocyte-like cells against the infiltration score. The Spearman correlation was computed with cor.test function in base R (33).

### Highly Interpretable Feature Extraction With Seg-SOM for Breast Cancer

The SOM interaction matrix is a summary statistic quantifying average spatial distances of nuclei from pairs of different classes. Formally, for a pair of segmented nuclei in an H&E image with indexes *i*, *j* and their respective planar positions in the image *v_i_*, *v_j_* ∈*R* ^2^, we define the nuclei distance using a Gaussian kernel with a standard deviation of 50 μm, as


$$d(i,j)=\exp(-||||v_{i}-v_{j}||||/2\cdot 50^{2}).$$

Interaction between classes *k* and *l* is then defined as the mean proximity between all pairs of nuclei in these classes, i.e.,


$$I(k,l)=\Sigma_{i,j}\;d(i,j)for\;i\in C(k),j\in C(l)$$

where C(k) and C(l) are sets of indices of nuclei in classes *k* and *l* respectively. For 49 classes in our study, the SOM interaction matrix is a 49×49 matrix with elements *I(k,l)*, where *k*, *l* ∈{1, 2,, … ,49}.

Some of the features in this interaction matrix show strong correlations between 0.8 and 0.9; hence, the entire interaction matrix is reduced *via* non-negative matrix factorization (NMF) to a smaller feature set of 100 features, where the maximum correlation between any two features is below 0.2.

To perform NMF, we used a sklearn.decomposition.NMF function in python with n_components=100. We used NMF (as opposed to PCA) as it forces all feature coefficients to be positive, and that aids with the interpretation of the results. As it is easier to interpret the sum of two features that are present on the analyzed picture compared to subtraction of two features with opposite signs.

We used five-fold cross-validation for the classification task on the IDC-negative and IDC-concurrent DCIS dataset. We chose a logistic regression model for the classification task due to the simplicity of the model and the high level of interpretability of the results, which often comes at the cost of classification performance. With logistic regression, one can easily obtain both the strength of a feature, as well as the directional effect of a particular feature indicating whether the feature is predicted to increase the probability of the outcome or decrease it. These qualities are either more challenging to interpret or absent in feature importance schemes for more powerful models such as tree-based forest or gradient-boosting models. First, we train a logistic regression classifier on the 100 features obtained with NMF with an L1 regularization weight of 0.1, resulting in sparse model coefficients. We next select the five features with the largest magnitude of coefficients. Subsequently, we use the five selected features to train a second logistic regression classifier on the same training dataset. We finally test the held-out data. The above procedure is repeated for 5,000 iterations, and we report the precision, recall, F1, and area-under-the-curve scores.

## Data Availability Statement

The code to run Seg-SOM is available under https://github.com/kidzik/som.

## Author Contributions

ŁK, EY, and MM conceived the study. EY and MM performed experiments and analyzed the data. KS wrote the cell segmentation code. ŁK and RW supervised the study. RW obtained funds to support the study. All authors contributed to the article and approved the submitted version.

## Conflict of Interest

KS was employed by Ground Truth Labs.

The remaining authors declare that the research was conducted in the absence of any commercial or financial relationships that could be construed as a potential conflict of interest.

## Publisher’s Note

All claims expressed in this article are solely those of the authors and do not necessarily represent those of their affiliated organizations, or those of the publisher, the editors and the reviewers. Any product that may be evaluated in this article, or claim that may be made by its manufacturer, is not guaranteed or endorsed by the publisher.

## Supplementary Material

Supplementary Figure 1 Distribution of immunoscore and SOM Lymphocyte class nuclei counts over different stages of breast cancer progression. **(A)** TIL infiltration assessed by a pathologist as an immunoscore (1-low, n = 155; 2-medium, n = 89; 3-high, n = 22), and **(B)** the log2 count of hierarchical SOM Lymphocyte class nuclei predicted by the Seg-SOM on breast progression dataset stratified by different stages of breast cancer progression: normal breast ducts (normal, n = 56), ducts with early neoplasia (EN, n = 62), ducts with ductal carcinoma *in situ* (DCIS, n = 58), and regions of invasive ductal carcinoma (IDC, n = 90).

Supplementary Figure 2 ROC curve with AUC from the logistic classification of IDC-positive *vs.* IDC-negative DCIS. The red line shows the receiver operating characteristic (ROC) curve and the averaged area-under-the-curve (AUC) of 5,000 iterations of the logistic regression model trained with five-fold cross-validation. TPR, True Positive Rate; FPR, False Positive Rate. The blue dotted shows the ROC curve for a random classifier, as a reference.

Supplementary Figure 3 Visualization of the four-dimensional SOM model. Fourteen Haralick texture features from three color channels were extracted from each nuclei image, showing an organization in which darker, smoother nuclei are located in the bottom left of the SOM grid. This can be described as a 4D SOM, in which each learned cell substate of the original SOM is further divided into a 7×7 space based on color and texture features, resulting in a grid of dimensions 7×7×7×7, where the texture and shape spaces are essentially disentangled. The gray boxes show the number of cells from the dataset falling within a particular node, and the yellow boxes are the ID numbers of each node.
