# Supplementary figures and images for: Self-Organizing Maps for Cellular In Silico Staining and Cell Substate Classification
Source: Front Immunol. 2021 Oct 29;12:765923. doi: 10.3389/fimmu.2021.765923 (PMC8588845; doi:10.3389/fimmu.2021.765923)

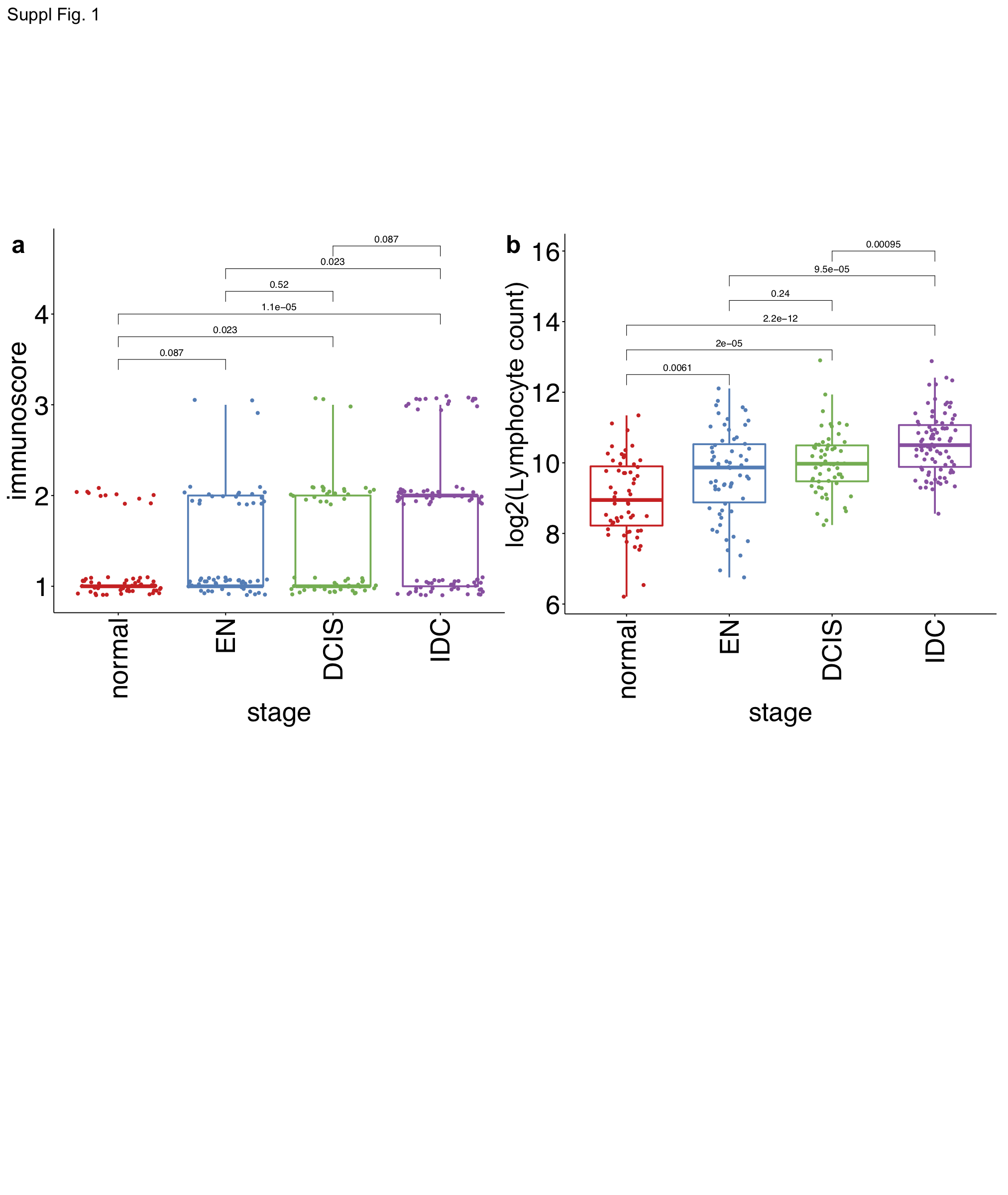

Supplement: Supplementary Figure 1 — Distribution of immunoscore and SOM Lymphocyte class nuclei counts over different stages of breast cancer progression. (A) TIL infiltration assessed by a pathologist as an immunoscore (1-low, n = 155; 2-medium, n = 89; 3-high, n = 22), and (B) the log2 count of hierarchical SOM Lymphocyte class nuclei predicted by the Seg-SOM on breast progression dataset stratified by different stages of breast cancer progression: normal breast ducts (normal, n = 56), ducts with early neoplasia (EN, n = 62), ducts with ductal carcinoma in situ (DCIS, n = 58), and regions of invasive ductal carcinoma (IDC, n = 90). [file Image_1.tiff]

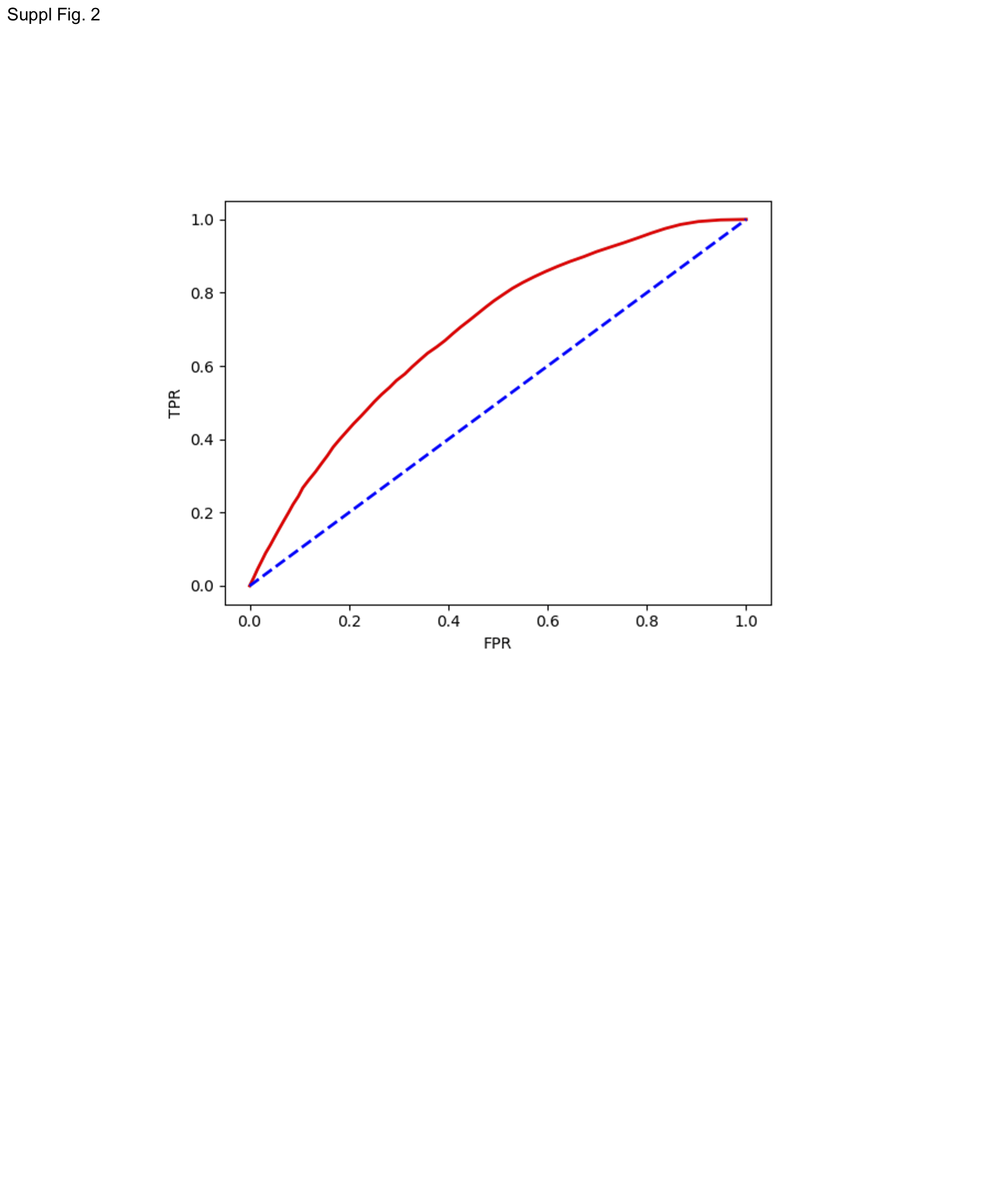

Supplement: Supplementary Figure 2 — ROC curve with AUC from the logistic classification of IDC-positive vs. IDC-negative DCIS. The red line shows the receiver operating characteristic (ROC) curve and the averaged area-under-the-curve (AUC) of 5,000 iterations of the logistic regression model trained with five-fold cross-validation. TPR, True Positive Rate; FPR, False Positive Rate. The blue dotted shows the ROC curve for a random classifier, as a reference. [file Image_2.tiff]

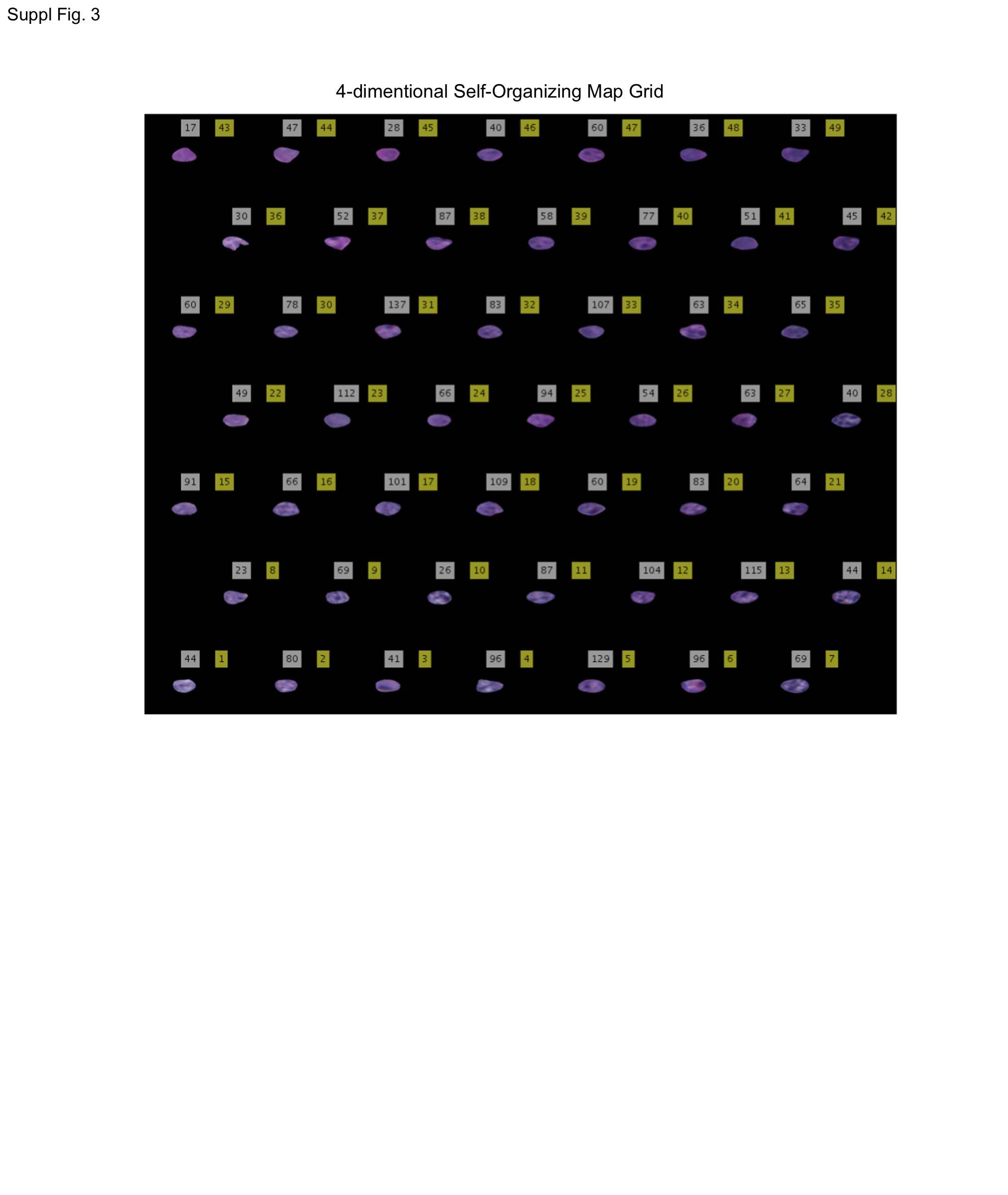

Supplement: Supplementary Figure 3 — Visualization of the four-dimensional SOM model. Fourteen Haralick texture features from three color channels were extracted from each nuclei image, showing an organization in which darker, smoother nuclei are located in the bottom left of the SOM grid. This can be described as a 4D SOM, in which each learned cell substate of the original SOM is further divided into a 7×7 space based on color and texture features, resulting in a grid of dimensions 7×7×7×7, where the texture and shape spaces are essentially disentangled. The gray boxes show the number of cells from the dataset falling within a particular node, and the yellow boxes are the ID numbers of each node. [file Image_3.tiff]
